# Future agriculture with minimized phosphorus losses to waters: Research needs and direction

**DOI:** 10.1007/s13280-014-0612-x

**Published:** 2015-02-15

**Authors:** Andrew N. Sharpley, Lars Bergström, Helena Aronsson, Marianne Bechmann, Carl H. Bolster, Katarina Börling, Faruk Djodjic, Helen P. Jarvie, Oscar F. Schoumans, Christian Stamm, Karin S. Tonderski, Barbro Ulén, Risto Uusitalo, Paul J. A. Withers

**Affiliations:** 1 Department of Crop, Soil and Environmental Sciences, University of Arkansas, Fayetteville, AR 72701 USA; 2 Department of Soil and Environment, Swedish University of Agricultural Sciences, P.O. Box 7014, 75007 Uppsala, Sweden; 3 Department of Soil and Environment, Bioforsk, Fred. A. Dahls vei 20, 1430 Aas, Norway; 4 USDA-ARS, 230 Bennett Ln., Bowling Green, KY 42104 USA; 5 Swedish Board of Agriculture, Dragarbrunnsgatan 35, 75320 Uppsala, Sweden; 6 Department of Aquatic Sciences and Assessment, Swedish University of Agricultural Sciences, P.O. Box 7050, 75007 Uppsala, Sweden; 7 Centre for Ecology & Hydrology, Wallingford, Oxfordshire OX10 8BB UK; 8 Alterra Wageningen UR, Alterra, P.O. Box 47, 6700 AA Wageningen, The Netherlands; 9 Environmental Chemistry, Eawag, Überlandstrasse 133, 8600 Dübendorf, Switzerland; 10 Department of Physics, Chemistry and Biology, Linköping University, 58183 Linköping, Sweden; 11 MTT Agrifood Research Finland, 31600 Jokioinen, Finland; 12 School of Environment, Natural Resources and Geography, Bangor University, Bangor, LL57 2DG UK

**Keywords:** Implementation, Manure, Mitigation measures, Monitoring, P management, Transport pathways

## Abstract

The series of papers in this issue of *AMBIO* represent technical presentations made at the 7th International Phosphorus Workshop (IPW7), held in September, 2013 in Uppsala, Sweden. At that meeting, the 150 delegates were involved in round table discussions on major, predetermined themes facing the management of agricultural phosphorus (P) for optimum production goals with minimal water quality impairment. The six themes were (1) P management in a changing world; (2) transport pathways of P from soil to water; (3) monitoring, modeling, and communication; (4) importance of manure and agricultural production systems for P management; (5) identification of appropriate mitigation measures for reduction of P loss; and (6) implementation of mitigation strategies to reduce P loss. This paper details the major challenges and research needs that were identified for each theme and identifies a future roadmap for catchment management that cost-effectively minimizes P loss from agricultural activities.

## Introduction

Phosphorus (P) impairment of surface waters remains a concern worldwide, such as in Asia (Wang 2006; Novotny et al. 2010; Dai et al. 2011; Sun et al. 2012; Li et al. 2015), Europe (Hilton et al. 2006; Withers and Jarvie 2008), South America (Shigaki et al. 2006), and USA (National Research Council 2008; Dubrovsky et al. 2010). Agriculture is a proven, but variable, contributor of P to many impaired waters (Sharpley et al. 2009; Ulén et al. 2010; Haygarth et al. 2012). Remedial strategies have been in place for 20–30 years to address these impairments, for example, in the Chesapeake Bay Watershed (U.S. Environmental Protection Agency 2010a), Mississippi River Basin (Dale et al. 2010), Florida’s inland and coastal waters (U.S. Environmental Protection Agency 2011), and Lake Erie Basin (Sharpley et al. 2012a). In many cases, however, water quality improvements have been less than expected for several reasons; these include but are not limited to legacy P inputs (i.e., P from prior land and nutrient management), climate fluctuations, ineffective conservation practices, and inadequate P management policies (Mulla et al. 2008; Meals et al. 2010; Sharpley et al. 2013; Jarvie et al. 2013a).

**Fig. 1:**
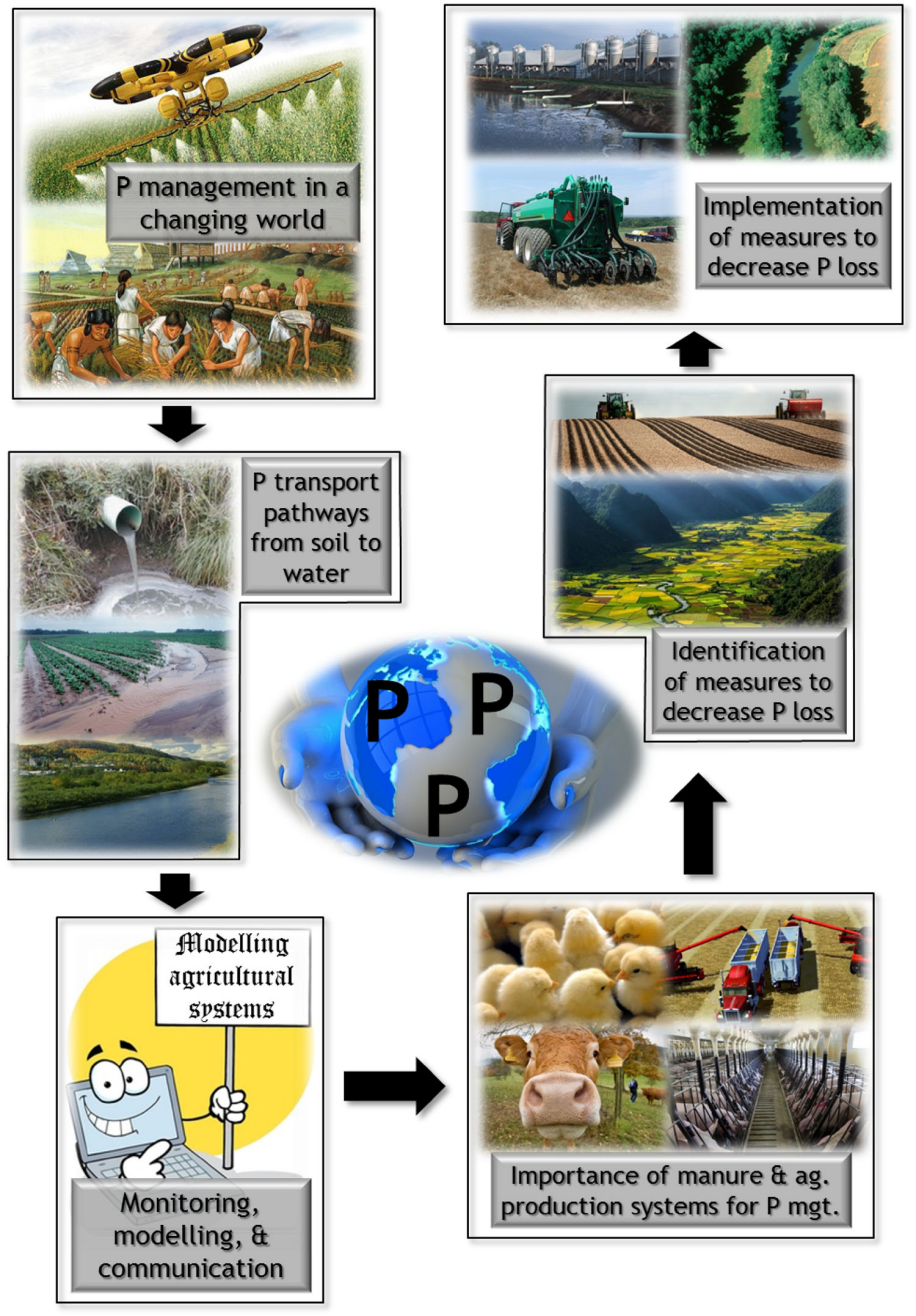
Conceptual model of the IPW7 synthesis of major research needs on agricultural P use and water quality

This continued water quality impairment provided the critical backdrop to the 7th International Phosphorus Workshop (IPW7) held in Uppsala, Sweden in early September, 2013. Major goals of this conference were to discuss current research on P management in agricultural systems and water quality impacts and to identify major gaps and future research needs. The latter objective was addressed by discussion groups focused on six scientific area themes (Table 1), within which questions were identified by conference attendees prior to the conference. The six scientific themes are depicted in Fig. 1 and are interrelated in the sustainable management of global P resources. Delegates met throughout the conference, and insights were gained as the conference proceeded. This paper summarizes the discussions and research recommendations.

**Table 1:** Synopsis of challenges and research needs identified by delegates at the 7th International Phosphorus Workshop, held in Uppsala, Sweden, September 2013

Theme	Challenges	Major research needs
1. P management in a changing world	Increasing P-use efficiency of diverse cropping systems, along with great water-use efficiency Fertilizers including mineral and organic sources Cost-effective recovery of P from manures and organic by-products and sludges Reconnecting spatially separated arable and livestock production systems	Crop breeding for increased P-use efficiency Development of 4*R* strategy to site-specific practices Unifying disparate policies to address P management and sustainability among countries Options for restructuring agriculture to the close P cycle
2. Transport pathways of P from soil to water	Magnitude and timescales over which P is retained and remobilized along transport pathways, and how this contributes to the accelerated storage of ‘legacy’ P within the landscape Quantifying subsurface water and P pathways and fluxes Evaluating processes and rates of P retention and recycling along transport pathways and up-scaling to the watershed Understanding long-term historical trajectories of legacy P accumulation and drawdown along transport pathways	Interfacing with digital terrain models, current GIS land use, soil surveys, and farmer knowledge of land response to identify drainage patterns Use of ‘background’ chemically inert tracers, already present in the environment, to evaluate hydrological pathways across watersheds Changing land use effects on P loss in surface and subsurface transport pathways Long-term monitoring of P loss pathways and fluxes along land–water continuum
3. Monitoring, modeling, and communication	P transport in subsurface drainage still poorly understood Model credibility can only be achieved with careful independent calibration, verification, and validation Models are increasingly used in policy decision-making, quickly providing maps and numbers at user low cost Monitoring is essential but costly Communicating model uncertainty and limitations to policy makers and public is the responsibility of the modeler	Monitoring programs must have clearly defined goals Long-term monitoring at various scales is essential All nutrient inputs and sources in catchments need to be represented Accurate models estimating P movement in artificial and preferential flow pathways Selection of the right model for the right scale and purpose Communication of model benefits and limitations is as important as predictions
4. The importance of manure and agricultural production systems for P management	Spatially disconnected intensive arable and livestock production systems exacerbate broken P cycle Vale of manure and other P-rich by-products inadequately recognized Development of cost-effective manure treatment and cost-beneficial by-products is currently limited Reduce urban waste generation, increase waste and by-product quality, and ensure recycling in agriculture	Plant genotype development and rhizosphere mgt. to stimulate P mobilization in low P soils Development of chemical and biological treatment that enhances fertilizer P value of generated by-products Assess possibilities of diversifying agricultural systems that sustain a closed P balance Overcoming the acceptability and biosecurity concerns of the public with using by-products as fertilizers
5. Identification of appropriate measures to decrease P losses	Edge-of-field P loss reductions brought about by conservation practices are highly site-specific High cost of conducting site-specific edge-of-field studies Disconnects between agricultural and limnological researchers limit cause and effect response development Still difficult to assign P levels in aquatic systems to current land use inputs or legacy inputs stored in soil and sediments	Innovative sampling and analytical technologies to make field assessment cheaper yet reliable at high sampling frequencies Development of amendments to sequester P in soil, manure, and by-products but retain plant availability Development of new cropping systems and rotations with great P-use efficiency, including catch crops
6. Implementation of measures to decrease P loss	Conservation measures can retain P on the land that will eventually become slow P release legacy sources Uncertainties of when and how much improvement in water quality will occur with restrictive land use of P Balancing the demand for cheap food with the desire for clean water Embracing the paradigm of adaptive system mgt. with stakeholder involvement, and flexible monitoring and policies Acceptance of green labels or sustainability metrics for environmentally sound-source food has been limited	Development of road map for equitable balance of restoring impaired waters with food security of increasing population that is more affluent Estimating the legacy of past land use and recovery pathways that are long and tortuous Given reversion to “pristine” conditions may not be possible, what aquatic environments are achievable and affordable? Targeting the right remedial measures at the right level to be cost-effective, with or without cost-sharing

## Theme 1: management in a changing world

### The main challenge

Improvements in agriculture in the last 50 years have dramatically increased grain and protein production in a very cost-effective manner (Fig. 1). For example, a tripling of global agricultural output in the last 50 years enabled food to become more abundant and cheaper, with agricultural prices falling about 1 % a year between 1900 and 2010, despite an increase in the world’s population from 1.7 to nearly 7.0 billion (Fuglie et al. 2012; Ball et al. 2014). Specialization and fragmentation of arable and animal production systems, however, have brought new pressures to bear on agricultural management within catchments. Historically, catchments generally had a sustainable nutrient balance. More recently, however, large amounts of nutrients removed, either as inputs (fertilizer and feed products) or outputs (meat and produce) on a national, regional, or global scale, which brings new pressures, challenges, and therefore, calls for new solutions (Cordell et al. 2009). Global complexities and interdependencies in P balancing are exemplified by the fact that increased grain and animal production in Brazil is making inroads into traditional U.S. markets and U.S. producers supply a large percent of the meat consumed in Japan, as water quality constraints in Japan limit cost-effective production there.

Several reviews and status perspectives of global cycling of P have acknowledged that the biogeochemical P cycle is broken at global, national, regional, and farm scales (Elser and Bennett 2011; Sharpley and Jarvie 2012; Jarvie et al. 2013b; Ulrich et al. 2013; Haygarth et al. 2014). The broken cycle of P from mining, fertilizer production, and land application of P in livestock manure, human waste, and food waste is the underlying problem limiting sustainable P use. Contributing to a solution is the need to realign the inputs of P, reuse P from manures and residuals (Bonvin et al. 2015; Stutter 2015), recover P from waste, redefine systems, and reduce P losses (the 5*R*’s of P; see also Schoumans et al. 2015; Withers et al. 2015). This is an update of “4*R*” nutrient management stewardship (right form, right time, right place, right amount; International Fertilizer Association 2009; International Plant Nutrition Institute 2014), specifically to address the broken P cycle, which is the root cause of P-related use impairments of water from accelerated eutrophication. Thus, increasing the efficiency of P use within agricultural systems that include intensively concentrated and spatially separated livestock and arable operations is critical for closing the broken P cycle (Fig. 1).

### Increased P-use efficiency

At a cropping system level, management strategies available to improve overall P-use efficiency of cropping systems include the use of diverse crop rotations, the presence of cover or catch crops, and crop breeding for higher internal P-use efficiency, which will enhance crop P acquisition strategies. However, plant and microbial strategies need to be further improved, particularly the uptake of residual soil P (from applied fertilizers and manures) and subsoil P uptake (Richardson et al. 2011). At a fertilizer P-use level, the development and use of slow release fertilizers (including testing of products from pyrolysis of P-rich materials), mixing mineral and organic P fertilizers, fertigation, and the advancement of biofertilizers (i.e., inoculation of seed with effective P-solubilizing microorganisms), are important research questions. Applying P in the right form, right time, right place, and right amount is also vital, and one important question is how to enhance the availability of solubilized residual soil P without increasing P loss by leaching or runoff.

At a livestock system level, delegates identified the need to reduce feed P imports, encourage lower P animal diets that will not impact production goals, and encourage the addition of phytase to feed to make dietary phytate available to livestock. The high cost of recovering P from manure, in terms of the expense of equipment, infrastructure, and recurring chemicals remains a severe limitation to P recovery for most individual farm operations. Thus, there is an immediate need for the development of cost-efficient methods for P recovery from manure and organic by-products and sludges (e.g., separation-drying-pelleting, chemical extraction) but most are still too expensive relative to the value of the fertilizer product. Some technologies need up-scaling to improve cost-efficiency; each technology must avoid introducing other contaminants in treated by-products; others have legislative and attitudinal barriers to overcome.

Increased P-use efficiency comes at a cost, however. A label for sustainable production system, green labels, and environmental stewardship metrics could support higher food prices. However, the devil is in the details of program operation, to ensure that the metrics are defined, quantified, certified, and routinely verified.

### Climate change

The increasing concerns of limited crop production, brought about by climate change, have direct and immediate consequences for food security. However, this is not explicitly a P issue, but more a water availability and use efficiency issue, which also has important implications for plant P uptake and P-use efficiency. More reliable predictors of the relationships among water use, plant growth, and P uptake are needed to assess the potential impacts of changes in rainfall distribution on P-use efficiency. However, there is a need to increase water-use efficiency and management strategies in Southern Europe through a combination of water storage, new irrigation techniques, and controlled drainage systems, and to optimize P management accordingly. Additionally, desalinization becomes even more important, together with changes in crop types and plant species and varieties, to enhance water and P-use efficiency where possible.

Changes in rainfall will likely affect runoff patterns, river flows, and the mobilization and transport of P. Increased soil and water temperatures will affect chemical reaction kinetics and microbial activities, which control the cycling and release of P along the land–water continuum (Whitehead et al. 2009). Also, shifts to more frequent extreme rainfall events may potentially impact P transfers and loss. In the Lake Erie Basin, 33 % more rain in intense events in the spring since 2009 than in the preceding 10 years (10 % of rain fell March, April, May) have led to the increased P runoff and contributed to the increased extent and intensity of algal blooms in the lake (Joosse and Baker 2011; Sharpley et al. 2012b; Smith et al. 2014). Options to promote resilience to climate change include improved water management strategies that limit losses of P and recycle water on arable land at the same time, methods to increase soil infiltration capacity, and methods to strengthen soil aggregates against dissolution (e.g., structured liming, application of organic matter, and adopting land practices that enhance organic matter build-up).

### Policy interventions

Conference delegates identified the main challenges to P management as P imbalances at global and local scales, with P surplus in intensive livestock production regions, and the general disconnect of livestock production from areas of arable production where feed concentrates are produced. It was further concluded that low P-use efficiency by crops can contribute to the accumulation of residual P in soils. It was clear that demands for relatively inexpensive food and global markets are the main drivers of P transfers, disconnects, and losses which have broken the P cycle. Although it is clear that cheap food is needed in some parts of the world, the question was raised whether cheap food is what we really need in all cases. It was also pointed out that cheap food would not be cheap, if societal and environmental costs of unsustainable productions systems were accounted for and passed on to the consumer. Whilst policy interventions exist, they vary greatly from country to country, such that there is no clear, coherent roadmap for future changes in production systems and P cycles.

There is also an increasing call for a more serious debate about the structure of agriculture; should we centralize animal breeding and production even more to make P recovery from manure viable, or should we strive for decentralization of animal production to facilitate manure P application at rates that are aligned with crop needs? In a similar vein of system restructuring, the reduction in meat consumption, e.g., by introducing tax on meat has long been discussed as a means of decreasing the demand for concentrating livestock production and associated P surpluses with respect to local crop needs. In Sweden, for instance, reducing meat consumption has been debated in relation to both climate change and P runoff. In the Swedish general elections of September 2014, the debate transitioned to the possible adoption of a meat tax as a policy intervention to reduce meat consumption. In contrast, a recent study emphasized the need to facilitate informed public choices and presented a consumer meat guide where four environmental impact indicators were used in a life cycle approach (Röös et al. 2014). Several research needs associated with making environmental information comprehensive to the public were identified.

## Theme 2: transport pathways of P from soil to water

### The main challenge

When P is land applied in the form of either mineral or organic fertilizer, surface and subsurface hydrological pathways transport P from the land surface, where it is a valuable resource used to achieve and maintain optimum plant yields, to receiving waters where it can be a major contributor of water-use impairment (Kleinman et al. 2009). Hydrological pathways along which P is transported are often complex and tortuous, involving multiple contributions from surface and subsurface transfers (Haygarth and Jarvis 1998a; Heathwaite and Dils 2000) (Fig. 1). It is difficult to make a reliable a priori assessment of whether transport and partitioning processes known from the literature are relevant for a particular catchment (Holländer et al. 2009). Educated guesses can be provided based on available basic information (e.g., topography, climate, soils) if this information suggests sufficient similarity with known cases. Such predictions can be improved by increasing the local knowledge based on different sources (e.g., farmers, geophysical information). However, not all factors and processes may be known from the literature and past experience may have serious biases.

The complexity of subsurface drainage and difficulties in deconvoluting flow pathways were identified as a key gap in our understanding of P transport and fate, particularly in groundwater-dominated watersheds (e.g., Jarvie et al. 2014), and during mixing of groundwater and surface waters during the hyporheic zone transport (e.g., Lapworth et al. 2011). Moreover, it was recognized that hydrological pathways along which P is transmitted do not simply transport P conservatively, but act as a series of reactive conduits, mediating P flux transformations through retention and recycling of P, on a range of timescales from years to centuries (Jarvie et al. 2012) (Fig. 1).

Short-term P retention along transport pathways may help protect downstream receiving waters from the acute effects of high P loads, providing an important ecosystem service, particularly in headwater streams (Hoellein et al. 2012). However, longer-term re-release of stored P can provide a chronic source of ‘legacy’ P. A fundamental research challenge is, therefore, to gain a better understanding of the magnitude and timescales over which P is retained and remobilized along transport pathways, how this impacts downstream receiving waters, and how this contributes to storage and release of ‘legacy’ P within the landscape (Sharpley et al. 2013; Lehtoranta et al. 2015).

### Research needs

#### Quantifying surface and subsurface pathways and flows

Challenges in measuring subsurface water flows, pathways, and retention times are currently a major barrier to quantifying accurate fluxes of P in subsurface (especially deep flow/groundwater) pathways. For modeling P transport pathways at the field scale, there is often a lack of knowledge about drainage patterns and locations of artificial drainage and other preferential flow pathways (Gentry et al. 2007). Whilst it was felt that many major transport pathways have been researched, several pathways were mentioned as being under-researched. Examples are the hyporheic zone, flow and nutrient flow paths in karst regions, wind erosion, phosphine gas (from rice paddies), leaching from unmanaged septic systems, flash flooding in Mediterranean climates, and cattle grazed in dry creek beds that flood during the rainy season. To address these shortfalls, research opportunities include

- Tracer injections to explore pathways and water residence times at the field and hillslope scale. Moreover, ‘background’ chemically inert tracers, which are already present in the environment from hydrochemically distinct water sources, can provide information on hydrological pathways across a wider range of catchment sizes (Soulsby et al. 2004; Jarvie et al. 2014).
- Digital terrain models (e.g., Sonneveld et al. 2006) and GIS-based classifications of soil hydrology [e.g., the ‘Hydrology of Soil Types’ (UK HOST’) classification; Boorman et al. 1995; Hahn et al. 2013] offer a useful template, as a simplified spatial assessment for modeling water flow pathways at the catchment scale. Integrating the digital landscape of soil hydrology types with background tracer studies offers opportunities to understand pathways, residence times, and the hydrological functioning of catchments (Soulsby et al. 2006).
- Whilst geophysical techniques and high-resolution LIDAR topographic profiling also offer solutions, farmer knowledge often provides the key insights into subsurface hydrology (e.g., which locations of the field stay wet) for field-scale modeling of transport pathways (Djodjic and Villa 2015).

#### Measuring processes and rates of P retention and recycling along hydrological transport pathways

- Microbial processes are believed to play a major role in P cycling, but it is difficult and time consuming to measure these directly, and this may lead to misrepresentation of process controls and over-estimation of the role of abiotic (sorption) controls.
- Our understanding of the importance of different processes that control P flux transfers along pathways is constrained by the standardized methodologies we routinely employ. Indeed, our estimations of net P ‘adsorption/desorption’ using, for example, EPC_0_ measurements probably encompass a whole range of biotic as well as abiotic processes.
- We are also constrained by our ‘operational’ definition of P fractions. For instance, measurements of “dissolved reactive P” may include substantial colloidal and hydrolysable organic/polymeric P fractions, which have different sorption characteristics than phosphate ions, and may undergo differing microbial transformations and bulk soil measurements. They may therefore not adequately predict the chemistry of dispersible colloids (Liu et al. 2014).
- We need a better understanding of in situ reaction kinetics controlling P transformations along pathways. For example, we need to consider how representative batch equilibrium sorption experiments are given the changes in redox conditions, and disruption of microbial communities occur when samples are removed from the natural environment and taken back to the laboratory. As a consequence, there is a need for in situ measurements to evaluate P flux transformations and controls on P spiraling along surface and subsurface transport pathways.

## Theme 3: monitoring, modeling, and communication

### The main challenge

The U.S. Environmental Protection Agency (USEPA 2010a) considers non-point sources of sediment, nutrients, and pesticides as one of the leading causes of water quality impairments, as do most countries in Europe. By definition, non-point source contaminants are much harder to identify and thus, harder to manage than point sources. This is confounded by the fact that landscape hydrology is highly variable, both spatially and temporally.

Non-point source models represent mathematical descriptions, ranging from simple (risk assessment indices) to more complex scientific understanding about chemical, physical, and biological processes that influence both point and non-point source contaminant loads within a catchment (Fig. 1). In their most comprehensive form, models can integrate information over a catchment scale and suggest where beneficial management practices (BMPs) are most likely to decrease catchment-scale nutrient losses. Thus, use of non-point source models provides a method of simulating the risk of P loss or estimating actual losses including the relative effects of change in climate, land use, and land management practices on sediment and nutrient loadings from large complex catchments. As a result, models can quantify change to gage progress. Numerical ranking provides strong appeal to policy makers and managers; however, this appeal can sometimes bring false confidence and misconceptions (Boesch et al. 2001).

### Monitoring

Monitoring is critical to addressing the main objectives of non-point sources management strategies, and present unique challenges to reliably represent site-specific variations in time and space (Verheyen et al. 2015). Monitoring programs are designed to identify nutrient losses and their sources areas, quantify the effects of mitigation measures, and document conservation program effectiveness. However, there is a cumulative uncertainty associated with water quality monitoring. This uncertainty is derived from, for example, stream flow measurement, water sample collection frequency, sample preservation and storage, and analysis (Toor et al. 2008).

Water quality data must further be related to information on catchment characteristics (e.g., soil properties, drainage conditions, contribution from point sources) and on agricultural activities such as crops grown, fertilization regimes, and soil cultivation practices. Access to such data is crucial for the interpretation of water quality data. Thus, the inherent landscape and management characteristics of monitored catchments must be stated, so that they can be related to surrounding agricultural areas where less information on agricultural management and nutrient loads are available. This would improve the applicability of monitoring results for larger agricultural areas (Kyllmar et al. 2014).

Delegates defined the following specific research needs to improve monitoring of catchment processes and response to land use management changes:

- There is a need to define clear goals for monitoring (e.g., to evaluate impairment status or understand processes for a given system).
- Long-term monitoring is essential, which should include baseline, extreme, and representative sites. Also, it was suggested that a few selected sites should be intensively monitored in conjunction with a larger number of less intensively monitored sites. Whilst such long-term monitoring is critical, it should be sufficiently flexible enough to be adapted to new concerns and issues.
- Adequate long-term (>10 years) monitoring of catchments is essential to reliable model calibration; however, there is often a limited amount of long-term water quality data that would be sufficient to estimate P and sediment loads in streams (representative of storm and base flow). A well-distributed network of monitoring stations across all land uses, topographic conditions, and sub-catchments of the larger catchment would assist in model evaluation and verification when estimating at smaller scales.
- Long-term (at least decadal scale) catchment monitoring is needed to be able to reliably track lags and changes in legacy P contributions. Moreover, there are clearly opportunities to exploit existing historical datasets to explore the timescales over which ‘legacy’ P is stored and released (e.g., Haygarth et al. 2014).
- Legislation may be needed to ensure the continuity of long-term monitoring. For example, new conservation strategies in the U.S. that provide cost-share funds to farmers to implement practices, now require that 10 % of the funds be allocated to monitoring the effectiveness of those practices (U.S. Department of Agriculture, Natural Resource Conservation Service 2014). Additionally, that same agency has established a standard for monitoring the quality of edge-of-field surface runoff and sample analysis, which must be followed for program eligibility (U.S. Department of Agriculture, Natural Resources Conservation Service 2012a, b).
- New developments with sensors that allow high resolution and continuous, real-time monitoring of certain components, such as nitrate, turbidity, dissolved oxygen, and electrical conductivity can help elucidate processes governing P release, transport, and biological impacts on receiving waters.
- Monitoring at different scales is needed, with field, farm catchment, and basin scales all being important. At each scale, detailed information on farm management and soil information is needed.

### Modeling

It is of critical importance that model developers clearly define what the model is useful for and what it is not designed to do. Likewise, users must decide what they want to accomplish with a model. For example, one must consider the scale (field, catchment, or basin), time (flow event, annual, or multi-year), and level of accuracy (0.1 or 10 kg ha^−1^ year^−1^) that needs to be simulated, as well as the amount and quality of data available. Model uncertainty arises due to an imperfect representation of the physics, chemistry, and biology of the real world; numerical approximations of the governing equations; inaccurate model parameter estimates; and uncertainties in model input data (Harmel et al. 2006). Sources of model uncertainty are often interrelated and further complicating matters is the observation that multiple models and parameter sets may provide similar predictions of a given dataset (Beven 2006). Nevertheless, because models play an important role in scientific studies and public policy decision-making, every effort must be made to obtain reasonable estimates of model uncertainties (National Research Council 2008). When insufficient knowledge is available on the error distributions of model inputs and parameters, reasonable estimates can often be based on values reported in the literature or on professional judgment of the modelers (Haan et al. 1998; Harmel et al. 2010; Bolster and Vadas 2013).

Standardized methods to quantify this uncertainty involve forcing the model to “fit” historically measured data, if available, with predetermined limits of performance (Harmel et al. 2010). This will assist modelers in quantifying the “quality” of monitoring data for calibration and verification and will assist in determining model accuracy, and evaluating model performance. Whenever possible, the uncertainty should be represented in the model output (e.g., as a mean plus a standard deviation) or as confidence limits on the output of a time series of concentrations or flows. Knowledge of the cause and effects of uncertainty, as well as the measurement of uncertainty, is more important than the best-fit model output in making “real-world” management decisions. Thus, it is incumbent on the modeler to explicitly express the assumptions made in representing the system which is being modeled. This will allow the user to assess how these assumptions affect the model outcome and may ultimately affect decisions based upon modeled results.

Despite such cautionary realities, the role of models will be more and more important over the next decade in making catchment management and policy decisions to identify critical source areas and target BMPs and evaluate effects of climate change. Thus, model evaluation and uncertainty is essential and should be clearly documented in any model development (Beven 2006; Reichert and Mieleitner 2009; Andersson et al. 2014). It is, therefore, critical that any use of non-point source models must be associated with data collection and monitoring to further improve process representation in models and to test model estimates (Oreskes et al. 1994; Jakeman et al. 2006).

Delegates identified the following shortcomings and research needs:

- Models for different scales have different purposes, and care must be taken to select from the numerous models available that is appropriate to the end-user needs, as well as model functioning.
- Better synchronization between monitoring and modeling efforts is urgently needed to overcome the common problem that some models can be developed and released based on limited calibration data (Sharpley et al. 2011).
- Models are either for research (assessment of relative differences and effects) or for management (quantification of losses). Care must be taken to not use the wrong model for the wrong reason, and they must be used within the boundaries around which they were designed.
- Some management models/tools may be simple and easy to handle. However, complex models, such as that used to assess land management and water quality response in the Chesapeake Bay Basin, can be extremely data needy (Linker et al. 1999; U.S. Environmental Protection Agency 2010b).
- Given the site-specific complexity of processes controlling P transport along surface and subsurface pathways, a key challenge lies in generalizing and scaling up from individual studies to other watersheds. For instance,
- How can generic process representation from field experiments (rates of P release etc.) be incorporated into catchment delivery models (Johnes and Hodgkinson 1998)?
- Rather than distributed models simulating individual pathways, there are other modeling approaches that consider distributions in water residence times and implications for legacy contaminant delivery and water storage legacies (e.g., Kirchner et al. 2000)
- Storage of P at different locations along transport pathways (and with different retention times) needs to be addressed to get a better handle on legacy P accumulation and release. At the moment, models are calibrated at the catchment outlet, but P could be retained for years or decades in transit along pathways (even longer if there are standing waters). This needs to be addressed to avoid targeting the wrong sources and pathways.

### Communication

The tendency described earlier for decision makers to “believe” models because of their presumed deterministic nature and “exact” form of output must be tempered by responsible use of the models, such that model computations or “estimates” are not over-sold or given more weight than they deserve (Boesch et al. 2001; Pappenberger and Beven 2006). Above all, model users should determine that model computations are “reasonable” with respect to providing output that is realistic and based on input parameters that are within accepted ranges. Modelers should use all available measurements and multiple levels of comparison to evaluate if model estimates are realistic.

In an assessment of the role of models in decision-making, Silberstein (2006) noted that models have been useful in defining our understanding of natural systems, as data processing and analysis aids, and scenario testing that can indicate relative effect of different catchment management actions on water quality. However, Silberstein (2006) went on to warn that a false sense of accuracy and definitive graphics can easily mask model limitations. A lack of clear communication of the established boundaries within which a model must be used, along with the need to collect field data will perpetuate the dangers of inappropriate model use and interpretation. With this in mind, delegates identified the following shortcomings and research needs:

- The right model should be chosen for the right scale and purpose, where uncertainty is clearly communicated to users.
- Educational and communication efforts are needed to communicate uncertainty issues to stakeholders, due to the huge uncertainty caused by the knowledge and understanding of the modeler.
- Results from monitoring and modeling may be over interpreted and can be taken out of context, especially with communication barriers between research models and managers.
- Results should be communicated in an easily digested format for stakeholders. Scientists rely on journal papers for information.
- Scientists need to communicate to stakeholders model benefits, limitations, what models are designed to simulate and not simulate.
- Some modeling communities have good experience with openness and transparency regarding model limitations such as uncertainties, extensive data parameterization needs, level of model accuracy, and model boundaries. This engages stakeholders and creates better mutual understanding of uncertainties as to what models can and cannot do. Transparency about complexity, uncertainty, and model limitations leads to shared responsibility.
- Model limitations often spring from the system’s complexity and are diverse. It is a crucial responsibility of scientists (modelers) to ‘educate’ regulators and policy makers on complexity.
- Accessibility of model tools and relevant background information, as well as user-friendliness of model platforms, can enhance communication. Also, interactive, web-based platforms should be better used to this end (Collentine et al. 2015).

## Theme 4: the importance of manure and agricultural production systems for P management

### The main challenge

As a consequence of the spatial separation of arable and animal production systems, fertilizer P is imported to areas of grain production (Fig. 1). Phosphorus in harvested grain and other plant material is then transported to areas of animal production, where inefficient animal utilization of P in feed (<30 % utilized) results in P excreted as manure. This has led to a large-scale, one-way transfer of P from grain-to animal-producing areas that crosses catchment and even national boundaries and has dramatically broadened the emphasis of catchment management strategies. In many cases, environmental risk assessment has defined critical areas where manure is required to be applied in amounts aligned with crop P need, taking legacy P into account (van Bochove et al. 2006; Buczko and Kuchenbuch 2007; Heckrath et al. 2008; Sharpley et al. 2012a, b).

As the intensity of animal production within a catchment increase, farm P surplus (input minus output) becomes greater, soil P levels increase from land application of manure, and the overall risk of P and N loss increase (Pote et al. 1996; Haygarth et al. 1998; Withers et al. 2003; Djodjic et al. 2005; Svanbäck et al. 2013).

### Research needs

Greater P recycling in general and a greater coordination of recycling at global, regional, local, and even farm levels are needed. For instance, in areas with large P accumulations in the soil, we need to focus on how to efficiently use this P, which in some cases might be by reducing P inputs to less than that removed in crop and forage export for several decades, until soil P concentrations are reduced. Exploitation of P resources in soil by rhizosphere management, i.e., by manipulating rhizosphere (the thin layer of soil surrounding roots) chemistry and biology to increase P mobilization and acquisition and reducing the reliance on chemical fertilizer P may offer opportunities to improve P utilization in low input systems (Richardson et al. 2011). Such opportunities could also include optimization of P inputs from chemical fertilizers, manures, wastes, or by-products that embrace the “4*R*” nutrient management approach, along with the use of different plant genotypes, and rhizosphere management strategies to stimulate P mobilization. Generally, the value of P in manures and in urban and industrial by-products needs full recognition and has to be appropriately accounted for in nutrient management schemes (Stutter 2015), which may require innovative integration of financial incentives or stricter regulations. Closing the P and other nutrient cycles is a fundamental question that needs to be answered locally as well as globally in order to increase P-use efficiency and meet stricter water quality standards and nutrient criteria, and produce cheap food in sustainable production systems.

More detailed needs identified are as follows:

- The question of closing the nutrient cycle is not just manure P driven. Other nutrients and carbon must be addressed in concert with P to avoid any indirect negative consequences.
- Acceptability and biosecurity of land-spreading human/industrial waste, as well as economics, political, and public concerns are barriers to development.
- Composting, vermi-composting, biogas generation, dewatering and pelletizing manure, as well as incineration and ash use are options that might indirectly address biosecurity issues.
- Increasing the adoption of toilets that separate liquids and solids at source and facilitate appropriate nutrient recycling to land (Bonvin et al. 2015).
- Cooperatives of farms with different specializations and sharing of specialized equipment to move toward a community P balance.
- Cover crops, catch crops, intercrops, and crop residue cover are important for a wide range of climatic conditions, even though their efficiency toward P management can be uncertain under cold climates. All these options should be adapted to site conditions to realize their full potential.
- Some key considerations are rhizosphere engineering (root exudates, organic acids, enzymes, mycorrhiza, bacteria inoculum etc.), root architecture, and biofertilizers (using living organisms to mobilize P in the soil).

## Theme 5: identification of appropriate measures to decrease P loss

### The main challenge

There are many BMPs that can be implemented over a wide range of scales to minimize the loss of P from agriculture to surface and ground waters (Fig. 1). Marginal abatement cost curves and cost-benefit ratios can be useful to identify best (most cost-efficient) measures in a given situation, as can a landscape analysis based on elevation maps and soil mapping data (e.g., Hahn et al. 2014). Available BMPs are commonly grouped into measures that seek to reduce P accumulation in soils (i.e., control at source), decrease P mobilization from the source areas (e.g., by soil management or soil amendments) and those that are applied along the transport route between the source and watercourses (Uusitalo et al. 2015) (e.g., P filters; Fig. 1).

### Farm input decisions

Carefully matching dietary P inputs to animal requirements can reduce the amount of P excreted by animals (Poulsen 2000; Valk et al. 2000). Implementing a carefully planned diet tailored to meet the specific P requirements of animals in each phase of their growth will minimize nutrient loss to the environment in feces, urine, and gases. Reducing farm inputs of P in animal feed is a very effective BMP that can contribute to bringing about a lasting decrease in P loss to the environment. In addition, BMPs that are designed to drawdown excess soil P reserves can contribute to achieving a P balance (Messiga et al. 2015). In fact, other nutrient management measures are generally aimed at decreasing the potential for P loss and are seen as short-term “band aids” and not long-term solutions.

### Source management

Controlling at source is often the most cost-efficient measure, because soils that leak substantial amounts of P are logically beyond the point where further P applications give any substantial yield increases. Amending such soils with additional P is waste of resources, if not always from a farmer’s point of view (when the intent is to dispose animal wastes), from the viewpoint of society at least. Careful nutrient management planning on a field-by-field and farm basis is a major component of any remedial action plan to minimize the risk of nutrient loss from agricultural lands. As discussed above, one of the most important farm measures may be to process the manure to facilitate a more even distribution in the landscape and reduce the excess supply to some fields.

### Transport management

Transport management refers to efforts to control the movement of P from soils to sensitive locations such as bodies of fresh water. Phosphorus loss from fields via surface runoff and erosion may be reduced by conservation tillage and crop residue management, terracing, contour tillage, and cover crops. Critical source area identification serves as an important basis for targeting fields and implementing edge-of-field remediation measures such as buffer strips, riparian zones, impoundments (e.g., settling basins), and wetlands. These practices tend to reduce rainfall impact on the soil surface, reduce runoff volume and velocity, and increase soil resistance to erosion. Control of P mobilization by soil management, such as conservation tillage practices, usually decreases the loss of particle-associated P but may increase those of dissolved P (Smith et al. 2015). Conservation tillage would be a method of choice on landscape positions prone to erosion, but on flat, non-eroding parts of a field, there might be more effective ways to bring about a decrease in P loss.

Chemical amendments have shown promising effects in P loss abatement (Uusitalo et al. 2015), and some measures can be real win–win solutions, e.g., structure liming (a mix of CaO and CaCO_3_), which both improves the soil structure and crop yield, and reduces P losses (Ulén and Etana 2014; Svanbäck et al. 2014). However, none of these measures should be relied on as the sole or primary practice to reduce P losses in agricultural runoff.

Transport of P from agricultural catchments depends to a large extent upon the coincidence of source (soil, crop, and management) and transport factors (runoff, erosion, and proximity to water course or body). Source factors relate to catchment areas with a high potential to contribute to P export. For P, source areas can be spatially confined and limited in extent, generally reflecting soil P status and fertilizer and manure P inputs (Gburek and Sharpley 1998; Pionke et al. 2000). However, spatial uncertainties may still be considerable (Hahn et al. 2013).

Filters placed in the landscape, on field margins or in streams, are by rule more expensive options than those closer to the source of P loading. They may occupy productive land (e.g., wider buffer zones), require construction work (constructed wetlands), or need special materials (e.g., reactive permeable barrier-type P sequesters) that, even if having a low unit cost (e.g., steel slags), are economical to use in the proximity of their production sites only. Filters also need variable levels of maintenance.

Edge-of-field measures can play a role in reducing the transport of P to aquatic ecosystems, but only when applied to critical source areas (CSAs). Identification of CSAs is a key first step and should serve as one of the most important inputs to decisions on where and what measures to implement. There are, however, still some research questions that need to be addressed to ensure efficient implementation of such measures:

- Despite all studies, information on the efficiency of buffer strips still needs to be improved. For instance, in northern, cooler climates, buffer strips can be less effective at retaining P than in more temperate climates, as decaying vegetation may release dissolved P in spring runoff (Uusi-Kämppä et al. 2012; Cade-Menun et al. 2013). Nutrients can also be released from standing stubble in conservation tillage with freeze–thaw cycles (Elliott 2013). Research is needed on how effective P-binding amendments to buffers can be and how long they can enhance P retention.
- Improved laboratory protocols and field-scale experiences for estimating the P retention potential of various P-binding materials are needed. In addition, we need a better understanding of the retention mechanisms and of conditions that may lead to desorption or solubilization of retained P. When using residual material from drinking water treatment or industrial operations, for example, the release of Al, other non-desired elements, or high alkalinity or acidity, into the environment may present a potential risk to the health of the receiving ecological communities.
- The addition of biochar to soil has been suggested to increase the soil nutrient retention capacity (Verheijen et al. 2010), but there are varied experiences, and we need more knowledge about the factors that determine whether there is a net positive effect on nutrient retention.
- There is a knowledge gap with respect to the factors affecting efficiency variations and cost of mitigation strategies based on P retention in ponds and wetlands (e.g., Kynkåånniemi et al. 2013; Beutel et al. 2014), and in particular if they rely on plant uptake and biomass harvesting as the main removal mechanism for dissolved P.
- Knowledge of P partitioning in loss pathways is, however, fundamental to enable policy makers to determine which mitigation policies might be most effective in terms of response time and reduction efficiency, leading to realization of further measures required or reducing expectation on the pace of change.

## Theme 6: implementation of measures to decrease P loss

### The main challenge

For real and lasting changes to occur in agricultural systems, balancing production and environmental stewardship constraints, there needs to be a greater consideration of socioeconomic drivers of what, how, and why some conservation practices are adopted and others are not (Kleinman et al. 2015) (Fig. 1). This leads to a greater emphasis on consumer-driven programs and education, rather than assuming that farmers will absorb the total costs associated with implementing remedial practices. Remembering that, except for decreasing off-farm import of P and increasing on-farm P-use efficiency, BMPs are only a temporary band-aid to minimizing the off-site transport of P and receiving water impacts. There needs to be a discussion of how regional as well as national agricultural infrastructures can control P inputs to farming systems and assess large-scale nutrient balances. For example, cost-share monies for confined animal feeding operations in northeastern U.S. catchments are now linked to farmers demonstrating that nutrient inputs to the farm are reduced by feeding animals at a level consistent with standardized dietary requirements of P. This exemplifies how public policy can address the source or cause of excess P concentrations, and how public investments can provide a long-term mechanism for overcoming infrastructural barriers.

### Research needs

- An open and forthright dialog on system response to P-based BMPs, the uncertainties involved, and the possible positive as well as negative consequences of management change among all vested stakeholders is needed. We are well aware of and have much documentation on the level to which P can accumulate in some intensive arable and livestock productions systems. The risk of water impairment is exacerbated by the fact that optimum levels of P for arable production can be an order of magnitude greater than needed for nuisance algal biomass proliferation. In fact, recent studies of the increased occurrence and severity of toxic algal blooms in Lake Erie suggest that a loss of less than 1 kg P ha^−1^ yr^−1^ can accelerate eutrophication (International Joint Commission 2013; Smith et al. 2014).
- There needs to be a discussion of the quality standards for river, lake, and estuarine environments that are achievable and affordable, given that pristine “reference” conditions are not achievable in some catchments with intensive agricultural production. Depending on the type of water body, the corresponding management target may be different (Stamm et al. 2014). Detailed cost-benefit analyses of P reduction strategies are needed to determine what is achievable, affordable, and even desired by the majority of catchment stakeholders. The EU’s Water Framework Directive requires “good ecological status” in terms of the ecological quality; only a slight departure from the biological community, which would be expected in conditions of minimal anthropogenic impact.
- Comparative studies are needed on policies across countries and how they work, for example, between countries within the EU. This will help address the question as to which is most efficient—the carrot or the stick, or probably a combination of both.
- A certification program for available BMPs is needed so that farmers can be better informed to decide what is best on a given farm in a given situation. Certification could be labeled on the products. For instance, Northern Ireland has a market-driven certification, which was initiated by supermarkets and gave good results in terms of environmental metrics, such as nutrient and water-use efficiency derived from life cycle analysis (Kloepffer 2008; United Nations Environment Programme 2012). However, are consumers prepared to pay more for products that are produced in an environmental friendly way?
- It must be remembered that some P loss cannot be avoided, due to events outside the control of a farmer, such as a large storm event. Thus, there needs to be an emphasis on quantifying background P losses in the context of defining achievable anthropogenic losses.

## Conclusions

Continued local, regional, and global water quality concerns have raised awareness of the need to identify landscapes and management practices that are more vulnerable to P loss and therefore at greater risk of impairing water quality further (Fig. 1). Given fiscal limitations, targeting conservation measurements for risky management on vulnerable landscapes is essential. This will require the appropriate use of data and model predictions derived at one scale (e.g., field or catchment) to address or target conservation measures at the same scale. Clearly, there are dangers in applying risk tools developed at, say, a field scale and applying them at a catchment level.

Even so, experience suggests that there should be a minimum level of conservation management that avoids risky practices on vulnerable landscapes. Further, in extreme cases of highly vulnerable landscapes, intensive agriculture itself may not be warranted, regardless of the suite of BMPs used or conservation measures adopted. Above a minimum level of conservation management, incentive- or reward-based programs could facilitate additional conservation strategies that protect water quality and ecosystem services.

Whatever strategies are implemented, they should be done in an adaptive manner because the complexities imparted by spatially variable landscapes, climate, and system response will require site-specific iterative solutions. Lag times for system response can also vary from a year to several decades, and this time generally increases as scale increases. At a field and farm level, research has demonstrated edge-of-field reduction in nutrient and sediment loss can occur within months of changing risky management. But the spatial complexity of catchment systems increases this response time for nutrients; for N, it is a function of mineralization of any stored organic pool and of residence time of groundwater flow pathways and for P, a function of slow release of legacy P stored in soils and fluvial sediments to more rapid surface and subsurface pathways (Lehtoranta et al. 2015). The significance of organic P mineralization is less clear.

Increasing water and sediment retention times along transport pathways can help enhance P retention whilst delivering multiple ecosystem benefits. For example, restoring headwater stream channels to a more natural hydromorphology increases water residence times and aquatic habitat diversity, which can promote greater nutrient assimilation. Such stream restorations also provide wildlife benefits, as well as enhancing wider aquatic ecosystem structure and function. It must be recognized, however, that a better understanding of ecosystem response to conservation measures is still essential to set reliable targets for restoration efforts. As we have shown, catchments are dynamic systems; post-implementation and conservation conditions on vulnerable landscapes will not be the same as pre-implementation conditions.

Finally, it is clear that, by necessity, models will be used to evaluate the potential for various management scenarios to mitigate water quality impacts associated with vulnerable landscapes. It must be remembered, however, that modeling is not a substitute for monitoring, which is essential to define, calibrate, and validate modeled scenarios.
